# Addressing the Cosmetic Resident Education Gap – a Junior Cosmetic Fellowship Program

**DOI:** 10.1093/asjof/ojae007.051

**Published:** 2024-04-12

**Authors:** Kelsey Lipman, Joshua Korman, Dung Nguyen

**Affiliations:** Stanford University, Palo Alto, CA; Stanford University, Palo Alto, CA; Stanford University, Palo Alto, CA

## Abstract

**Goals/Purpose:**

Incorporating adequate aesthetic surgery training into integrated plastic surgery programs in the United States has remained a challenge for several decades.^[1-4]^ Specifically, residents report low confidence in performing facial cosmetic procedures compared to breast and body contouring.^[1-2]^ This becomes increasingly relevant in the setting of heightened specialty creep and the rise in demand for cosmetic procedures overall according to the 2022 ASPS procedural statistics which show a 19% increase in cosmetic surgery procedures compared to the pre-pandemic 2019 report.^[5]^ In a survey of 257 residents, 26.4% felt confident performing a lower blepharoplasty, 25% performing a facelift, 16.5% performing an endoscopic brow lift, and 14% performing a rhinoplasty.^[2]^ This resident-reported difficulty with facial cosmetic procedures has shown little progress over time in reported survey data despite an increase in minimum cosmetic case numbers and length of training. A comprehensive aesthetic training also includes exposure to non-surgical interventions such as neuromodulators, injectables, non-surgical body contouring and facial rejuvenation technology. Though non-invasive fat reduction (ie. cryolypolysis) and non-surgical skin tightening (ie. radiofrequency micro-needling) have increased in demand since 2019 (77% and 22%, respectively), residents rarely have hands-on exposure to these treatment modalities.^[5]^ Several integrated plastic surgery programs have a dedicated year of professional development or research, providing an opportunity for programs to fill this gap between cosmetic surgery training and real-world demand.

**Methods/Technique:**

A junior cosmetic fellowship curriculum was created at a single institution focused on increasing confidence in performing aesthetic surgery, exposure to non-surgical cosmetic procedures, and increasing exposure to the business side of private practice. The fellowship was designed for a single resident during the professional development year of integrated plastic surgery training, completed between the third and fourth clinical years. The junior fellow spent the year within a single, multi-office private aesthetics practice in both the surgical and non-surgical setting. Over the course of the first four months (July 2023 - October 2023), the clinical experience of the Stanford junior cosmetic fellow was queried. Analysis of surgical case volume and non-surgical patient load was performed. This was then used to project surgical cases anticipated to be completed by year end. This was compared to case log minimums for graduating integrated plastic surgery residents in the United States, an essential metric of assessing resident experience and competence. Financial analysis of the non-surgical treatments by the junior fellow was performed. Using the initial four-month data, projections were also estimated to determine profitability for the practice over the course of the year-long fellowship.

**Results/Complications:**

Over the span of the initial four months, the junior cosmetic fellow completed 69 cases, 58% of which were breast / body and 42% of which were focused on facial rejuvenation. It is estimated that at completion of the fellowship, this projects to 207 cases, exceeding the cosmetic ACGME program requirements for graduation. Total sales generated by the junior fellow during the first four months were as follows: $22,802, $90,257, $78,563, and $75,084 USD. Therefore, total sales by year end are estimated to be $800,118 in non-surgical treatments – demonstrating expertise of the junior fellow in non-surgical procedures and also profitability for the practice.

**Conclusion:**

Increasing resident comfort with aesthetic surgical and non-surgical procedures is imperative as the demand for cosmetics continues to rise. Here we highlight the creation of a junior cosmetic fellowship, designed for programs with a year of professional development in their plastic surgery curriculum, to bridge the gap of cosmetic resident education. This junior fellowship model increases overall resident comfort in aesthetics and experience with facial procedures, a known weakness in resident training. Also, the addition of a junior fellow has shown to be financially lucrative for a practice, encouraging private practice attendings to consider this teaching model. Beyond the scope of programs with a dedicated year for professional development, this fellowship model may encourage embedded fellowships or incorporating mini-fellowships of 3-6 months in aesthetic surgery into residency programs outside of the traditional chief resident cosmetic experience.
